# Studies of the Crystallization and Dissolution of Individual Suspended Sodium Chloride Aerosol Particles

**DOI:** 10.1021/acs.jpca.4c02158

**Published:** 2024-05-21

**Authors:** Natalie C. Armstrong Green, Allen E. Haddrell, Florence K.A. Gregson, David Lewis, Tanya Church, Jonathan P. Reid

**Affiliations:** † School of Chemistry, University of Bristol, Bristol BS8 1TS, U.K.; ‡ Chippenham Research Centre, Chiesi Limited, Chippenham, Wiltshire SN14 0AB, U.K.

## Abstract

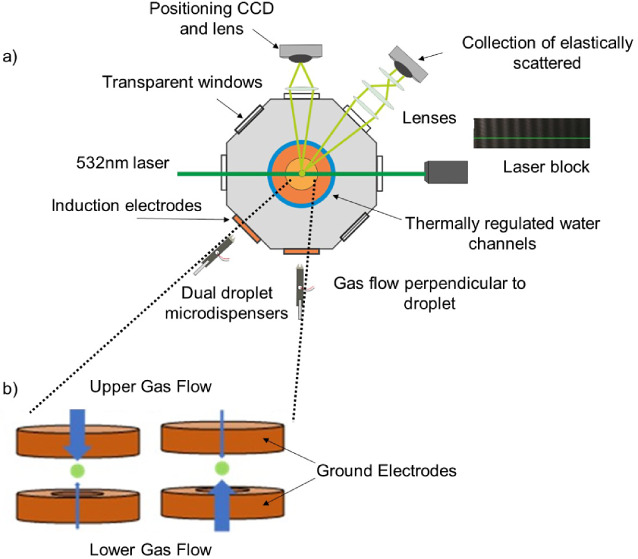

Aerosols transform between physical phases, as they respond to variations in environmental conditions. There are many industries that depend on these dynamic processes of crystallization and dissolution. Here, a single particle technique (an electrodynamic balance) is used to explore the crystallization and dissolution dynamics of a model system, sodium chloride. The physical and environmental factors that influence the dynamics of crystal formation from a saline droplet (whose initial radius is ∼25 μm) and the kinetics of water adsorption onto dried particles are examined. The drying relative humidity (RH) is shown to impact the physical properties of the dried particle. When a saline droplet is injected into an airflow at an RH close to the efflorescence RH (ERH, 45%), an individual single crystal forms. By contrast, when a compositionally equivalent saline droplet is injected into dry air (RH ∼ 0%), a salt crystal made of multiple crystalline particles is formed. Subsequent to crystallization, the crystal shape, morphology, and surface area were all found to affect the dissolution dynamics of the dried particle. Additionally, we report that the difference between the deliquesce RH and environmental RH significantly impacts the dissolution time scale.

## Introduction

The presence of water in air influences the physical and chemical properties of aerosol particles,^1^ impacting their physical state, size, and chemical structure.^2^ Spray or freeze-drying techniques are often used to produce dried particles from solution droplets ready for human consumption, most commonly in the food and pharmaceutical industries.^3^ The process of spray drying can form dry particles of different forms; depending on the solute properties, rapid water evaporation can form amorphous particles, whereas slower evaporation can lead to the formation of an energetically more stable crystalline form.^4^ Amorphous particles are seen as an ideal formulation when rapid dissolution is required, often used in pharmaceutics for devices such as dry powder inhalers (DPIs).^5^ However, spray drying conditions can also be selected to produce crystalline particles, which have slower dissolution kinetics and are more resilient formulations in humid environments.^6^ Crystalline aerosol particle often shows slow dissolution once a threshold relative humidity (RH) is achieved, with an induction time as water first adsorbs onto a particle surface.^4^ The dissolution of solid particles in the aerosol phase, i.e., the uptake of water vapor by dried particles to form a solution droplet, has implications for the efficacy of drug delivery to the lungs.^5,6^ The deposited fraction, physical state, and location of particles delivered to the lungs that are dependent on particle size influence the pharmacokinetics.^7^ For example, the bioavailability of an active pharmaceutical ingredient (API) within a DPI aerosol will depend on the degree to which it has dissolved in water; the degree to which the API dissolves during inhalation (prior to deposition) is unclear and in need of further study. Thus, a greater understanding and control over the dissolution kinetics of solid particles during inhalation may afford opportunities to improve the efficacy of an inhaled aerosolized formulation.^8^ Although there has been much research regarding the condensation kinetics of water on amorphous aerosol particles,^2^ there is limited literature on the condensation and dissolution kinetics of crystalline aerosol particles in a humid environment.^9^

The equilibrium humidity response of a crystalline aerosol system is governed by the efflorescence–deliquescence cycle, which is distinct for different chemical systems. Efflorescence, the formation of a crystal on dehumidification and drying, occurs when the water activity in the solution droplet phase falls below a certain critical value and the solute can no longer be sustained in a metastable solution. Deliquescence, transformation from solid to an aqueous solution phase, occurs when water molecules adsorb onto the surface of a solid until complete dissolution is achieved; this process occurs at a well-defined RH dependent on the substance and equal to the water activity in a saturated solution of solute. An aqueous sodium chloride droplet crystallizes once the RH is lowered to less than or equal to 45%, and a NaCl crystal deliquesces when the RH is raised to 75%. When the ambient RH is below the deliquescence relative humidity (DRH), water interacts with the solid particle via adsorption. A crystalline sodium chloride particle can absorb 2–3 monolayers of water at an RH < DRH before bulk dissolution is seen.^10^ As the RH increases, more water adsorbs onto the solid surface via capillary condensation until the RH of the gas phase is higher than the DRH, at which point a solute saturated film forms around the solid. The film is the basis of the continued deliquescence and water condensation process as it has a lower vapor pressure than that of pure water or the partial pressure of water in the gas phase. The water in the saturated film has a lower thermodynamic activity relative to water in the gas phase, providing the driving force for continued water condensation onto the particle until complete dissolution and equilibration with the water activity, RH, of the gas phase.^10^

Aerosol originating from a DPI starting formulation can have an exceedingly high deliquescence point (>90% RH).^11^ A list of common compounds is shown in Table 1.^12,13^

**Table 1:** Compounds and Their Associated DRH^12^

compound	DRH, 25 °C
EADD	>95
lactose	95
mannitol	96
ascorbic acid	95
fumaric acid	95
salbutamol sulfate	92

Once inhaled, the maximum RH the DPI aerosol will experience will be ∼99.5%, which may be near the deliquescence RH of the aerosol. Thus, in order to understand the degree to which a DPI aerosol will take up water/dissolve on the time scale of a single inhalation event (∼10 s), the relationship between dissolution rate with particle structure and RH (relative to deliquescence RH) must be considered. Here, we investigate factors that affect the dissolution rate of NaCl at or near the deliquescence point, including the RH, the crystal size and mass, and the crystal morphology. The aim of this work is to explore how RH influences the crystallization processes of a well understood inorganic aerosol and the subsequent effect on particle dissolution. Control of dissolution is shown to be possible using a single particle measuring technique, and the dissolution kinetics of a well characterized crystalline aerosol particle, sodium chloride, can then be explored.

## Methods

A single particle instrument, the comparative kinetic electrodynamic balance (CK-EDB),^14^ is used to infer the time-dependent size and morphology^15^ of individual aerosol particles while the RH and temperature of the surrounding gas phase are accurately controlled. The crystallization and dissolution kinetics of sodium chloride particles are explored.

### Comparative Kinetic Electrodynamic Balance

**Figure 1:**
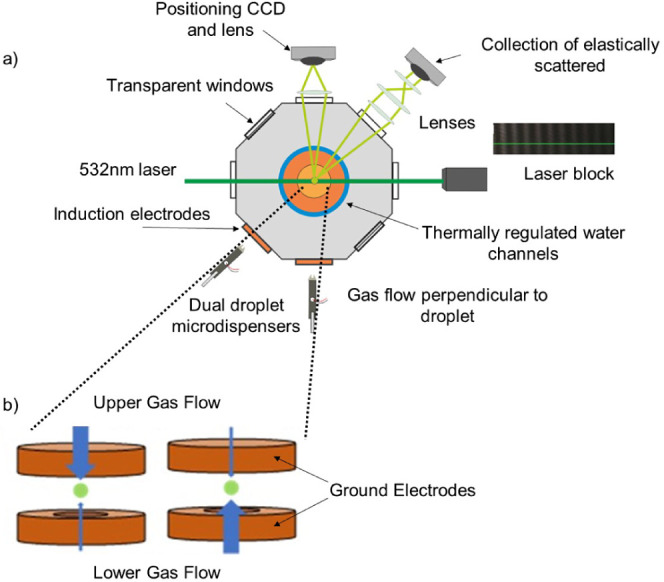
(a) An electrodynamic balance is used to measure the efflorescence and deliquescence dynamics of sodium chloride. The droplet/particle is probed by a laser light, where the scattered light from the droplet is collected as a phase function, which can be used to accurately estimate the size/phase of droplet/particle. (b) The RH the droplet experiences at the center of the trap can be switched between two gas flows (blue arrows) set to different RHs within 0.4 s.

A schematic of the CK-EDB is shown in Figure 1a. A brief description of the instrument will suffice here, and an extensive description can be found in the literature.^15,16^ The starting formulation of ∼0.17 mass fraction of solute NaCl in pure water is loaded into the reservoir of a microdispenser (Microfab MJ-ABP-01). The microdispenser produced individual droplets with an initial radius of ∼25–30 μm. During generation, a charge is imparted on the droplet by an induction electrode situated a few millimeters in front of the orifice tip of the microdispenser. The momentum of the charged droplet carries it to the central region of an electrodynamic field that is formed between two sets of vertically aligned concentric cylindrical electrodes.^17^ A DC offset applied to an AC field generated between the upper and lower electrodes is applied to the lower electrode, which counteracts the downward force of gravity acting on the trapped droplet.^17^

The temperature inside the trap is controlled by a mixture of water and ethylene glycol, which passes through a thermostatic water bath (F32-ME, Julabo). The solution flows through the mounting plates for the ground electrodes, thereby setting the temperature of the gas phase that flows through them. The thermostatic water bath is set to room temperature, 293 K, for all experiments described here. The temperature of the gas that passes over the droplets is the same as that of the ground electrode, which is measured continuously throughout the experiment using a temperature input device (USB-TC01, National Instruments).

The RH surrounding the levitated droplet is controlled by mixing two gas flows, one of humidified nitrogen and a second of dry nitrogen. The RH of the gas flow is estimated by measuring the evaporation rate of a pure water droplet and comparing the rate to a model;^18^ this results in an error in the RH estimate of ±0.1%. Two separate flows (each with their own set of RH) pass through the upper and lower electrodes. Mass flow controllers (MKS 1179A Mass-Flow, rated for 500 sccm N_2_) allow rapid switching (<0.4 s) between the upper and lower gas flows (Figure 1b). Since the droplet only experiences the higher velocity air flow, the rapid switch in air flows results in the droplets experiencing a change in relative humidity in <0.4 s;^19^ the RH of the chamber is not changed, rather just the microenvironment surrounding the levitated droplet. This rapid switch in RH can replicate the change in conditions during an everyday process such as inhalation, where an drug formulation travels from ambient air into humid conditions such as the lungs. To simulate the case of drug inhalation, the RH can be switched from <10% RH to 95% RH; thus, the rate of RH change that the particle experiences is similar to that during inhalation, but the RH of the airflows remains lower than that in the lungs (∼99.5%). The RH of each gas flow can be inferred from the evaporation rate of pure water droplets injected immediately before and after the salt droplet crystallization/dissolution experiment; based on the evaporation rate of the droplet and the temperature of the air flow, the relative humidity can be calculated.^19^

Once the particle is trapped, it is illuminated with a green laser (λ = 532 nm), and the elastically scattered light is collected in the form of an angularly resolved phase function. A geometric optics approximation is used to estimate the radius as a function of time.^15^ The radius estimate is accurate only for homogeneous, spherical droplets as they produce uniformly spaced fringes across the viewing angular range of ∼26°, centered at a viewing angle of 45°. However, when the droplet contains inclusions (e.g., particles suspended in a liquid droplet during the early stages of crystallization), the light scattering intensity variation with angle becomes irregular. It remains possible to estimate the droplet size as the angular separation between the peaks still reflects the host liquid droplet size.^14^ It is not possible to estimate the size of crystalline nonspherical particles (geometric approximation results in extremely noisy estimate of droplet size). For crystalline nonspherical particles formed from a solution droplet (such as NaCl), the dry radius can be estimated based upon the initial droplet size, solute concentration, and solute density:
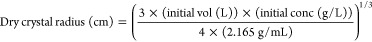
1

The geometric approximation, used to retrieve the radius from the fringe separation, requires knowledge of the refractive index (RI) of the droplet.^16^ In previous work to infer aerosol hygroscopicity^15,20^ and measure evaporation kinetics,^21^ we have shown that a RI correction based on ideal mixing can be adequate for correcting the radius data retrieved from the CK-EDB. The RI correction method accounts for a changing RI as the droplet solution becomes more concentrated upon evaporation or dilute on condensation. In this work, the droplet radius data are retrieved assuming a RI of pure water (1.3331), and we have chosen not to correct the estimated radius to account for a changing RI. This results in an error in the droplet radius, depending on solute composition, between 0% and ∼4%.

### Measuring Droplet/Particle Structure in Real Time in a CK-EDB

The connections between particle crystallization dynamics and subsequent particle dissolution are explored. First, studies of the crystallization of sodium chloride droplets are reported, followed by the dissolution kinetics of the same dried particles under varying environmental conditions. The capability provided by the EDB to rapidly change the RH in the gas phase from very low RH (e.g., dry) to very high (>90% RH) allows measurements of crystallization and then dissolution of the same particle.^14^ The ability to rapidly change the conditions the droplet experiences is unique to the CK-EDB; more established techniques that trap individual droplets in a chamber (e.g., optical tweezers) require tens of seconds to minutes for the conditions the droplet experiences to change.^22^ The dependence of the dissolution and condensation rates on the initial drying conditions, crystal surface area, and dried particle morphology can be studied. In order to explore these connections, the ability to differentiate between droplet and particle structures using the light scattered by the levitated particles must be exploited.

**Figure 2:**
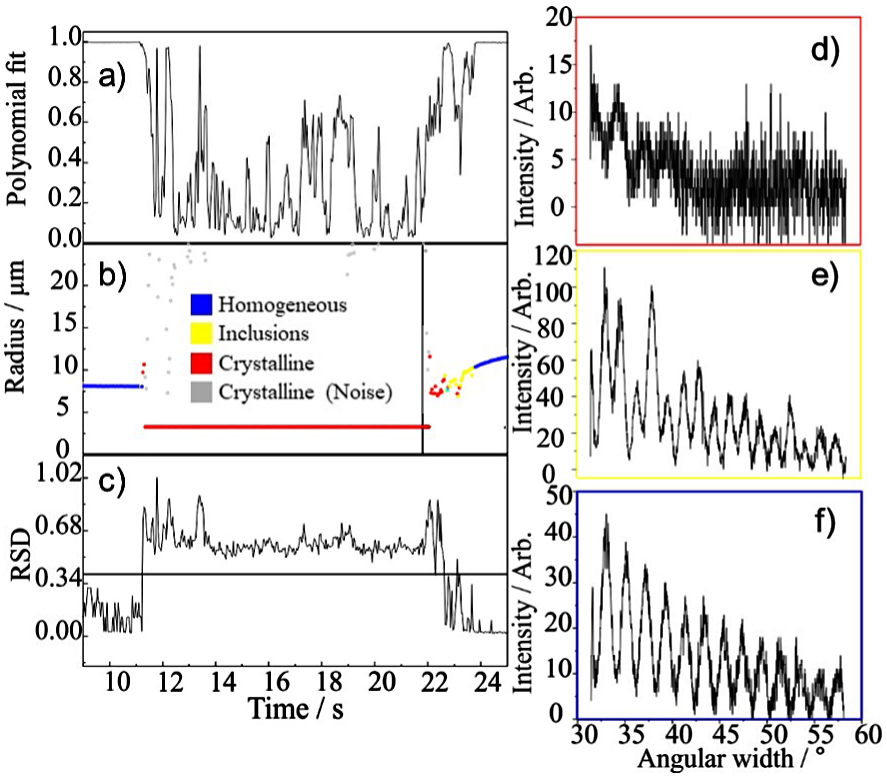
Correlation coefficient of the polynomial fit (a) and RSD value (c) for all phase functions during the lifetime of a droplet of a given radius (b) are used to distinguish between particle phases. The phase functions in the red, yellow, and blue boxes are examples of the scattering from a crystalline (d), inclusion (e), and spherical homogeneous (f) particle phases, respectively. Above an RSD value of 0.35 (horizontal black line), the particle is characterized as nonhomogeneous and nonspherical. When the particle is in this state, it cannot be sized via light scatter, which results in the reported radius being noise (“Crystalline (Noise)” in (b)). Once the RH is increased from dry to humidified (90% RH) at the time indicated by the vertical black line, ∼ 20.9 s, in panel, the particle transitions from crystalline to a droplet containing inclusions and finally to homogeneous.

The distinct character of the light scattering profile can be used to characterize the physical state of a levitated particle in the CK-EDB (see typical examples of the angular resolved phase functions in Figure 2). Four phase states have distinct scattering patterns: spherical/homogeneous droplet, a droplet containing inclusions, a droplet with a core–shell/radial concentration gradient, and a nonspherical particle.^15^ The time of crystallization is identified by a transition between identifiable scattering fringes and a noisy irregular pattern characteristic of a nonspherical particle. A thorough description of the method of phase analysis using light scattering can be found in the literature, based on three quantities to evaluate the character of the light scattering pattern.^15^ Briefly, the regularity in the angular separation between the peaks in the phase function is expressed in terms of relative standard deviation (RSD) of the average angular separation.^15^ Next, the angles of the fringe maxima are fitted to sixth-order polynomial and quadratic curves, yielding two correlation coefficients. As an example, the time-dependence of the sixth-order polynomial fit, RSD value, and retrieved radius are presented for a single particle in Figure 2. Consistent with our previous work, which examined over one million phase functions recorded from particles of a variety of known morphologies, if the RSD value is greater than 0.35, then the particle can be characterized as inhomogeneous.^15^ The correlation coefficient for the sixth-order polynomial fit can be used to determine when the particle transitions from an inclusion droplet to a spherical homogeneous droplet or vice versa. Note between ∼22 and ∼24 s, the RSD rapidly drops while the polynomial fit remains low; during this time, the structure is identified as a droplet containing inclusions.^16^

### Collecting a Dried Sample for Microscopic Imaging Using a Falling Droplet Column

To further explore the morphology of the dried particles, a falling droplet column (FDC) was used to generate and collect dried sodium chloride crystals. The FDC is a glass column with a square cross-section of height ∼50 cm.^12^ The same microdispenser is used for droplet generation on the CK-EDB and FDC, the initial sizes of droplets generated on the two instruments are the same, and the environmental conditions are consistent. Dried particles are produced at a temperature of 293 K and under either dry (0%) or ambient (∼45%) conditions. The RH is measured using a probe that is placed at the bottom of the column and is controlled using only a dry air flow, which is either on or off: 0% or 45%. The droplets that fall through the column deposit and are collected on a glass slide, which is then kept in a desiccator before imagining within the vacuum of a scanning electron microscope (SEM) (Joel IT300 SEM). Given that no additional forces beyond gravity are accelerating the particles when they deposit onto the glass slide (i.e., they are falling at a terminal settling velocity), the assumption is made that particles maintained their structural integrity upon deposition; the high reproducibility in observed particle structure supports this assumption.

## Results and Discussion

### Increase in Water Evaporation Rate During the Crystallization of Sodium Chloride and the Final Dry Particle Morphology

**Figure 3:**
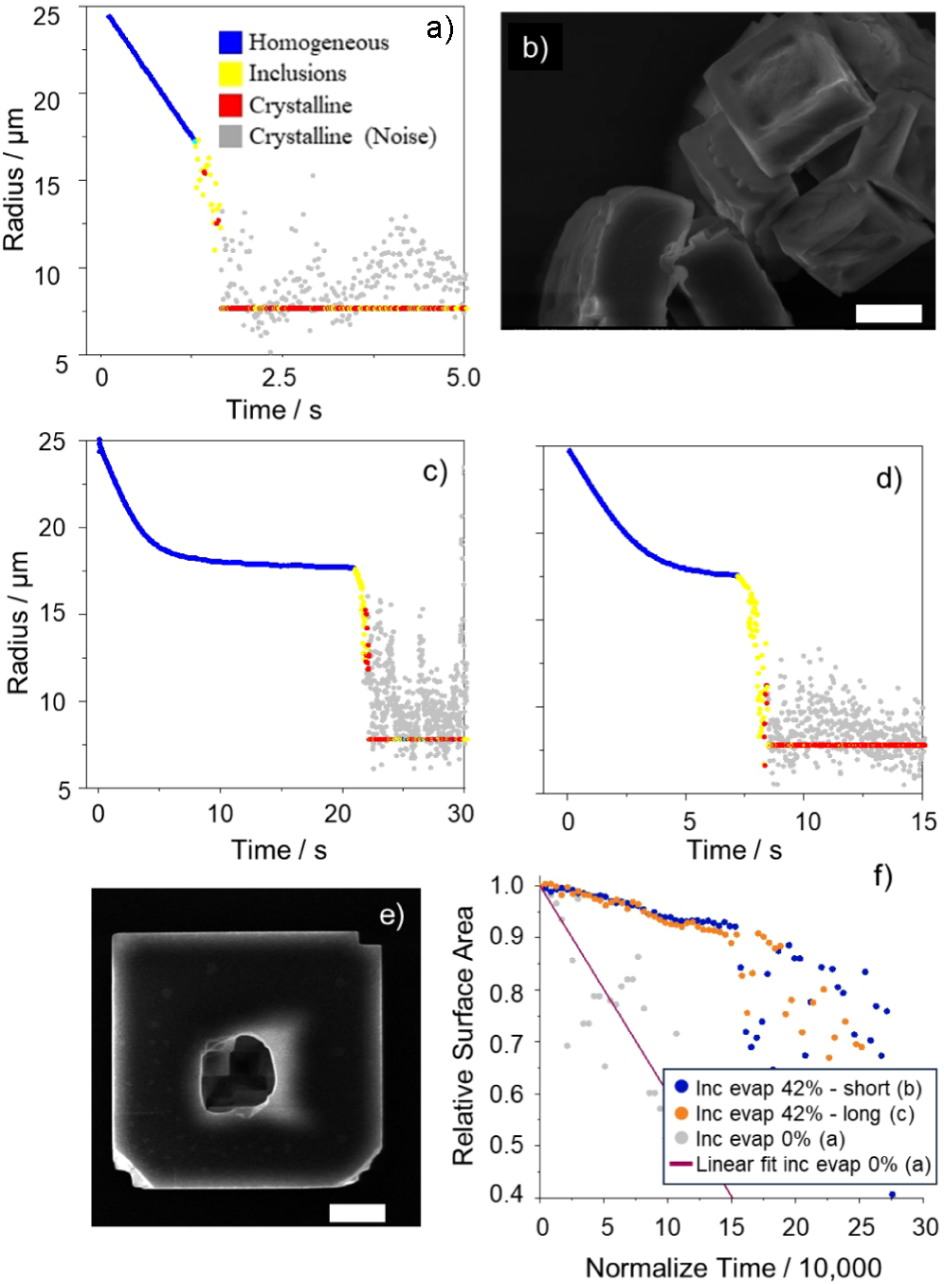
Crystallization dynamics of a solution containing sodium chloride and water at RHs of <5% (a) and 40% ((c) and (d)). Crystallization time at 40% RH varied, as shown in (c) and (d). (f) Evaporation rate of the droplet once inclusions are detected (Inc. evap), where *y* = *r*^2^/*r*0^2^ and *x* = *t*/*r*0^2^. SEM images of NaCl crystals formed from a saline droplet dried at RHs of 5% (b) and 40% (e); white bars indicate 5 μm.

Individual saline droplets of known composition were trapped in the EDB at RHs in the range from dry (2%) to the efflorescence RH (45%), leading to rapid droplet drying and crystallization. The angularly scattered light patterns were analyzed,^15^ and the phase of the particle was identified at times during evaporation as homogeneous, inclusion, or crystalline. Estimates of the dry radii of each particle, shown by the horizontal red lines in Figure 3, were calculated from the starting radius and solute concentration. While the estimation of the dry radius assumes a homogeneous spherical shape, an unlikely morphology for a sodium chloride crystal, it allows for relative comparison of dry sizes between all droplets. Additionally, the dry mass of sodium chloride can be estimated from the dry radius.

Saline droplets were exposed to different drying conditions, which led to varying evaporation rates, crystallization times, and crystal structures (Figure 3). In all environments drier than 45% RH (efflorescence RH of NaCl), the droplet surface recedes as water evaporates into the gas phase. At a critical supersaturation point achieved at the droplet surface (a concentration of twice the solubility limit), NaCl particles nucleate to form inclusions within the remaining solution.^14^ Eventually, the crystals grow as water continues to evaporate, and the suspension of undissolved crystalline particles merges to form a single dry particle. However, the time taken for a droplet to transform into a dried crystal depends on the environmental conditions. At an RH of 2% (Figure 3a), the water evaporation is most rapid, inclusions are formed within ∼2 s and a crystal within 2.5 s. However, at an RH of ∼40% (Figure 3c,d), i.e., close to efflorescence RH, the evaporation is much slower as the droplets first equilibrate with the gas phase moisture content, and a crystal spontaneously forms after some delayed time. It is important to note that the time taken from droplet generation through to crystallization at 40% RH was variable and ranged from ∼6 s (Figure 3d) to ∼20 s (Figure 3c). We have previously studied the evaporation rates and distribution of nucleation times, and this is not our focus here.^14^

The drying rate had a dramatic effect on the structure of the dry particles formed. When the drying conditions are set to 40% RH, it is more likely that crystallization is initiated through a single nucleation site, to be contrasted with the likely growth of crystals formed at multiple nucleation sites at lower RHs. At 40% RH, the final particles are single crystals, with nucleation and crystal growth of the first crystal nucleus to form (Figure 3e). By contrast, a polycrystal is formed during the rapid drying events that occur in dry air, as shown in Figure 3b. When the RH is close to 0%, the increased water evaporation rate leads to multiple nucleation sites, forming crystals that then grow competitively. Thus, this induces the formation of multiple crystals in the same droplet, described here as a polycrystal. The dry masses of the final dry crystalline NaCl particles are equal in Figure 3b,e.

Once nucleation occurs and crystal growth begins, the evaporation rate of water from the crystallizing droplet increases markedly (Figure 3f). The evaporation of water from the droplet during efflorescence is found to be dependent on RH, where the rate at 40% RH is slower than in dry air. The vapor pressures of water above the supersaturated sodium chloride aqueous surface just prior to and immediately after nucleation are approximately the same, and both evaporation rates are controlled by the diffusional gradient in the gas phase. However, water loss rates during the crystallization process are considerably higher and are likely a consequence of the elevated droplet temperature, resulting from the enthalpy released on crystallization. The rate of droplet size change, , is equal to
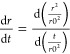
2where *r* and *t* are the radius and time, respectively. Near the efflorescence RH, a single large crystal is formed (Figure 3c–e), whereas, in dry air, multiple smaller crystals are formed simultaneously (Figure 3a,b). The evaporation rate of the homogeneous droplet into dry air (Figure 3a) prior to the start of crystal growth is equal to . After nucleation and the growth of crystal inclusions begin, the evaporation rate (from Figure 3f) is equal to . The enthalpy of crystallization for NaCl is the reverse of enthalpy of dissolution (Δ*H*_dissolution_ (NaCl) = +3.9 kJ mol^–1^). During crystallization in a finite volume droplet, where the internal conduction of heat is faster than conduction and convection into the gas phase, the droplet temperature increases (in this case by ∼ +8 °C). An increase in droplet temperature enhances the water evaporation rate by increasing the vapor pressure of water.^3^

### Condensation Profile during Dissolution of Dried Sodium Chloride Particles at 95% RH

Under “sink conditions”, a sufficient solvent is present for the solute to be fully dissolved. In a bulk solution phase, typically 5–10 times larger volume of media is used above the volume at which dissolution would be otherwise slowed.^5^ In the dissolution of aerosol, where water must condense from the gas phase, it is important to set the upper RH well above the DRH of NaCl (75%) when exploring dissolution dynamics, avoiding the limitation of water availability in the gas phase. The first step in exploring the time scale for dissolution of NaCl in the aerosol phase is to form crystals of a selected morphology and size in situ within the EDB by controlling the drying rate (Figure 3). Once formed, the dissolution of the same particle is studied by instantaneously switching the gas phase flow to 95% RH.^2,14^ After the switch, water adsorbs onto the crystal and dissolution begins, where complete deliquescence is categorized as the point when the aqueous sodium chloride droplet has become homogeneous. Transitions in the light scattering phase function from particle are used to estimate the times at which the particle is transformed in the phase.

**Figure 4:**
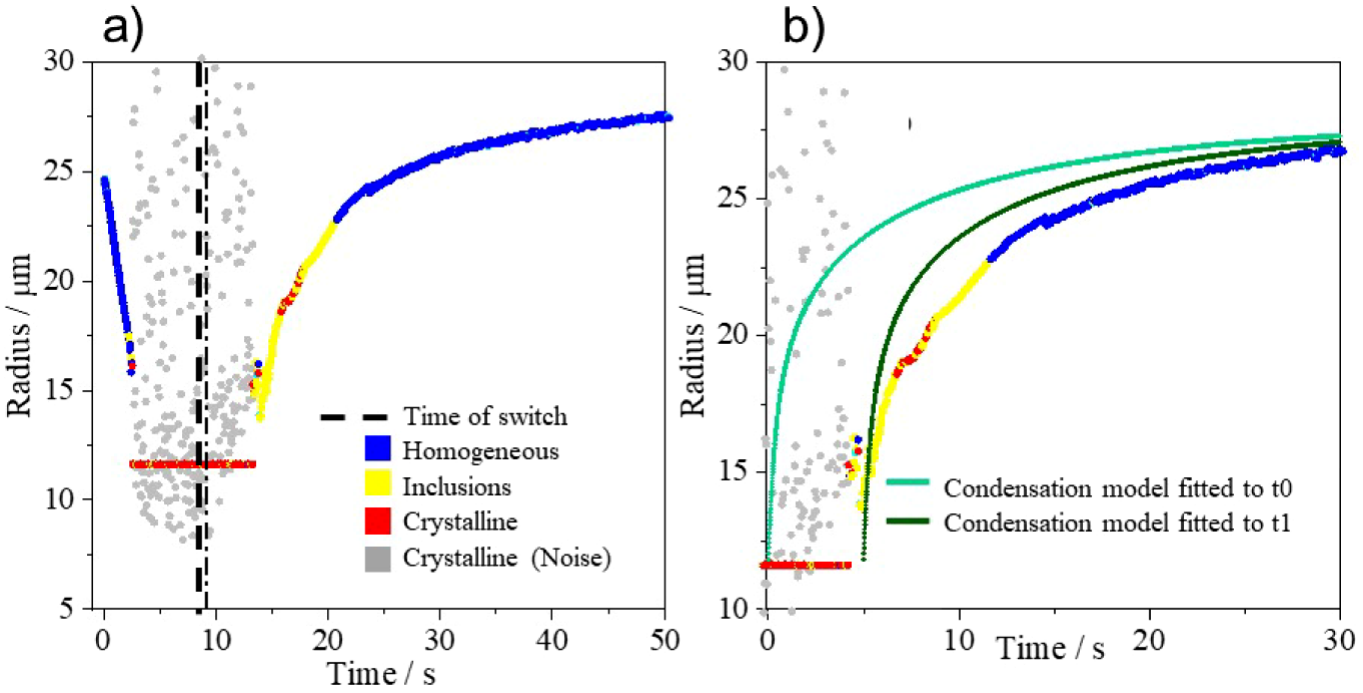
(a) Crystallization and dissolution of an aqueous droplet of sodium chloride that is trapped in dry air until crystallization, after which the RH is switched (time = dashed black line) to the upper flow, 95% RH. (b) Dissolution profile of a sodium chloride crystal compared with modeled simulations of condensation, where the rate is a solely gas-diffusion limited process.^23^ For comparison, the gas-kinetic model simulation is aligned at *t*0 (light green), the time of the switch, and at an offset time, *t*1 (dark green).

The complete crystallization and dissolution profile of a NaCl particle of initial aqueous solution concentration 171.2 g/L is shown in Figure 4a. A trapped aqueous NaCl droplet crystallizes in the EDB once the water activity of the droplet falls below 0.45 within ∼2 s. Approximately 5 s after crystallization, the RH is switched to the upper RH, ∼95%; the time of the switch is indicated by the black vertical line; the actual time taken for the RH to change to occur is ∼0.1 s.^14^ Once the RH is above 75%, above the DRH, water condenses onto the particle. After sufficient water adsorption, the crystal particle dissolves and forms an aqueous NaCl droplet.^23^ However, the scattered phase function from the particle indicates that it remains crystalline during an induction period of ∼5 s before any phase or size change is seen, referred to as a dissolution lag period below. After the dissolution lag period, the phase functions indicate that a droplet containing inclusions is formed.

Water molecules will adsorb onto the surface of an NaCl crystal particle before the RH is above or equal to 75%, the DRH. Below the DRH, water condensation leads to the formation of multilayers around the crystal particle in the range 50% < RH < 75%.^23^ Once the RH is above 75% detachment, and solubilization of ions can commence. Lanaro and Patey found that the initial stage is the detachment of the most exposed ions located on edges, i.e., corners or edges.^6^ The ions at corners and edges have the weakest bond energies so are more readily removed than ions on flat surfaces.^6^ At this point, a film made up of a saturated solution of NaCl forms around the surface of the particle.^12^ Vapor condensation and formation of a saturated film are the basis of deliquescence. The elevated gas phase RH continues to drive condensation, sustained by the gas phase concentration gradient, supplying water to the saturated film solution and supporting further dissolution. Once the edges and corners were consumed, the crystal became more spherical. From then on, the shape does not change until the final stage of dissolution.^6^ Langlet et al. produced a time sequence of environmental scanning electron microscopy (ESEM) images showing the dissolution of NaCl at RH > DRH.^24^ Additionally, Wise et al. observed water uptake on the surfaces of NaCl before the DRH.^25^

In our measurements, ultrafine solute inclusion particles become detached from the primary particle over time and are encompassed within an aqueous droplet. These inclusions continue to dissolve during the dissolution phase, and the inclusions persist for ∼7 s (Figure 4a), during which time the droplet size increases by more than 10 μm. During the phase in which the droplet contains inclusions, the uniformity in the scattering phase function returns, and estimates of droplet size can be made. However, it should be noted that sizing of a droplet containing inclusions is less accurate than a homogeneous, aqueous droplet.^15^ A homogeneous droplet is formed once sufficient water has condensed, and the fine particles have dissolved. The concentration of NaCl decreases until the water activity inside the droplet is equal to that of the gas phase, ∼ 0.95.^12^ Equilibration of water activity between the droplet and gas phase governs the final size of the particle. In Figure 4, the droplet equilibrates once the radius is ∼27 μm.

To better understand the kinetic limitations during the dissolution process, simulations from a condensation model^23^ (that includes the coupling of heat and mass transfer) are compared to experimental data (Figure 4b). The model is set with the same starting dry radius and final RH but does not account for dissolution dynamics, rather simply reflecting the transition in RH and gas-diffusion limited water transport. It is clear that there is a lag in the beginning of the condensation and particle growth. The simulation broadly captures the condensation rate when offset in time for closer comparison with the experimental data once condensation is clearly apparent. However, the model prediction is marginally faster and suggests that there are additional limitations to gas phase diffusion within the experimental data that are not captured by the model. The model assumes that the growing size is limited only by gas phase diffusion and heat transport and does not incorporate the additional temperature changes that result from crystal dissolution. The competition between the endothermic crystal dissolution and exothermic water condensation may account for the slower rate of droplet growth compared with that of the model.

### Dissolution Kinetics of a Dried Sodium Chloride Particle Is Highly Dependent on RH

**Figure 5:**
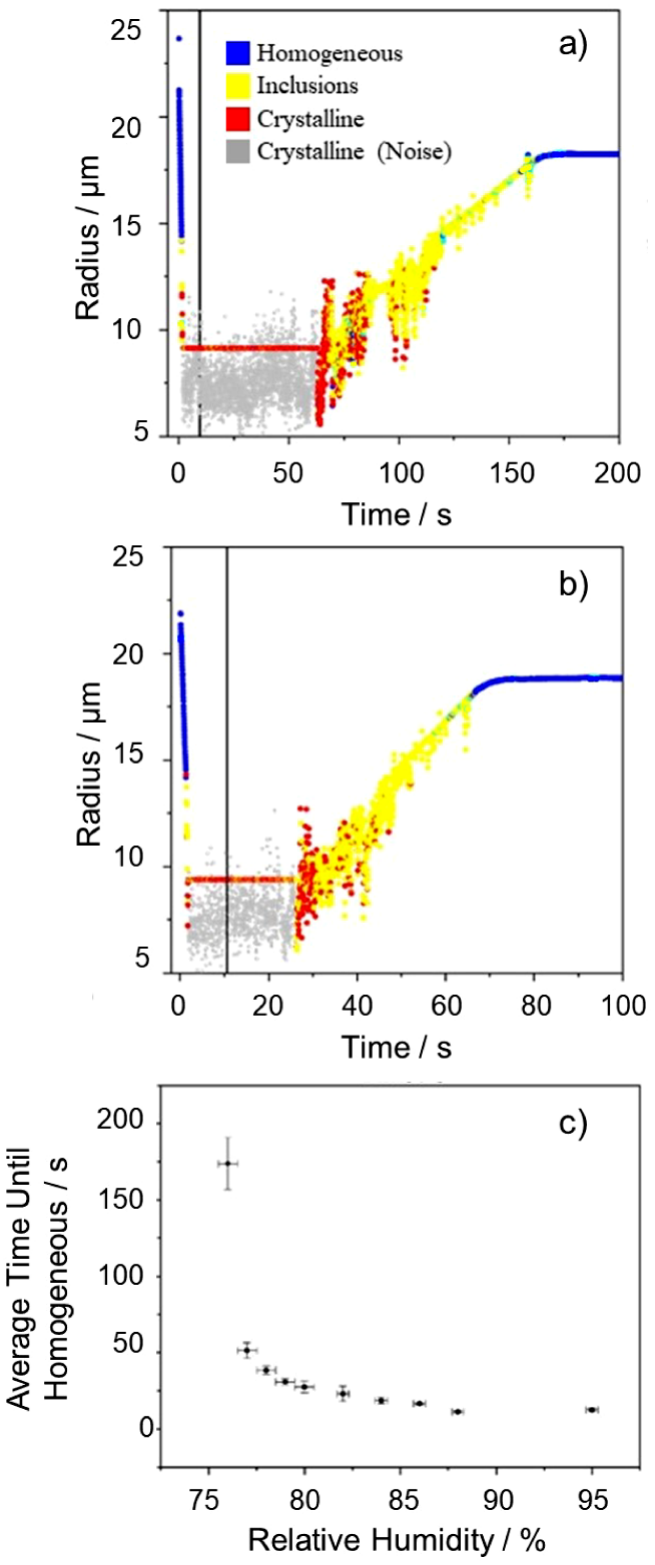
Exploring the dependence of dissolution dynamics of a crystalline NaCl particle on the water availability in the gas phase (RH), where all NaCl crystals were formed through drying NaCl droplets in 0% RH. Dissolution dynamics of a single NaCl particle at (a) 76% and (b) 78% RH, where the time that the RH is switched from 0% RH to either 76% or 78% is indicated by the vertical black line. (c) Average time taken for a NaCl crystal to become fully homogeneous as a function of RH. Each data point is an average of 15–20 dissolution profiles, where the RH was measured immediately before and after each dissolution measurement.

The rate of complete dissolution increases as the RH rises above the DRH.^12,26^ At RHs close to the DRH, the deliquescence process can be extremely slow,^12,26^ taking hours before the system reaches homogeneous equilibrium. Necessarily this will always be the case for poorly soluble active pharmaceutical ingredient particles when inhaled with high DRHs even at the high RHs of the lung, thus it is important to understand this process in the aerosol phase.^12^ To explore the degree to which a similar phenomenon occurs in the aerosol phase, the dissolution kinetics of particles at RHs close to the DRH (where the water activity at the solubility limit of the solute) is explored (Figure 5).

The time required for NaCl crystals to dissolve at varying RHs was performed 15–20 times at each dissolution RH, ranging from 76% to 95% RH. The average time until homogeneity was observed was assessed for each droplet. The time to reach homogeneity was estimated using the RSD and polynomial fit parameters discussed in Figure 2. The uncertainty in the RH-axis represents potential fluctuations in RH during the course of the dissolution measurement, which is ±0.5%. For each measurement (examples shown Figures 5a,b), a NaCl droplet of starting concentration 171.2 g/L was trapped and dried in the EDB at an RH of ∼0% to crystallization. The droplet crystallized within ∼2 s. After ∼10 s, the gas flow was switched to the elevated RH < 76% RH. The light scattering is then used to determine when the crystal had fully dissolved. Complete dissolution took ∼100 s at an RH of 76% (Figure 5a) and only ∼50 s at a RH of 78% (Figure 5b). A summary of the relationship between the RH and dissolution time is provided in Figure 5c.

A longer dissolution lag period at 76% RH indicates that the early stages of water adsorption and dissolution are the limiting factors governing the dissolution time. The difference between dissolution rates at 76% and 78% RH is the difference between the water activity at the surface of the crystal and at the droplet boundary, creating a significant water activity gradient. Once the RH reaches 88% then the dissolution lag time is constant, ∼ 8 s, driven by a gradient in water partial pressure in the near-particle gas phase that is a factor of more than 10 times that at 76–78%; additional increases in RH to >90% make relatively small changes to the gas phase gradient and the gas phase diffusion rate.

### Dependence of Dissolution Kinetics on Surface Area and Dry Crystal Mass

**Figure 6:**
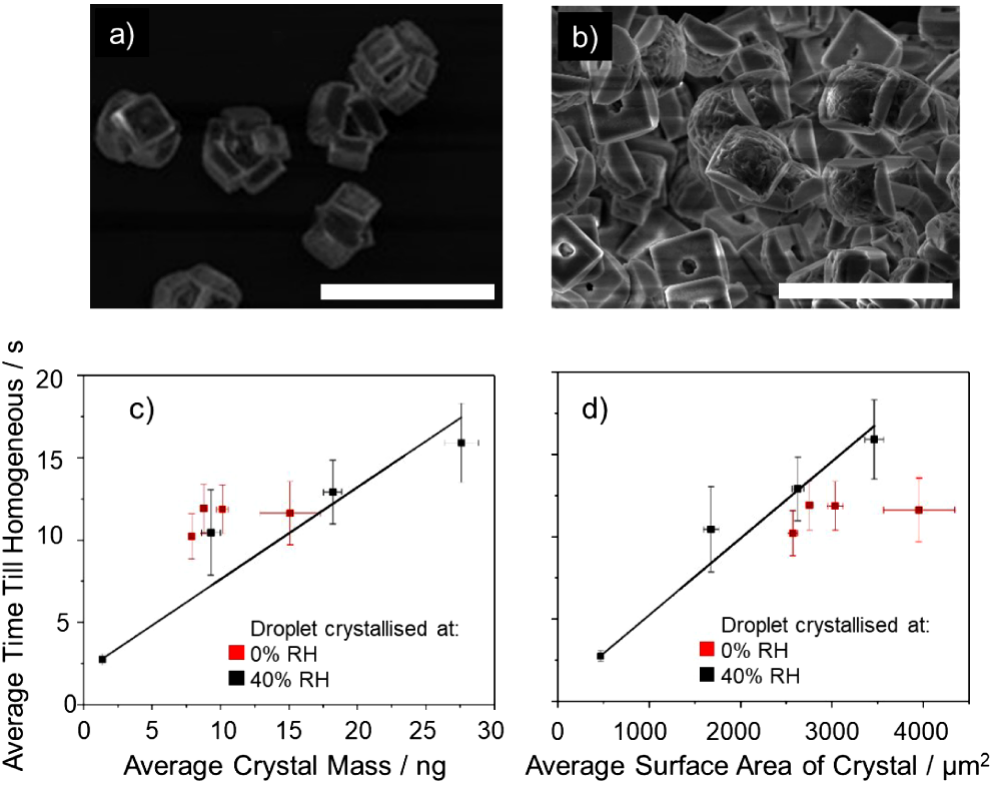
SEM images of sodium chloride mono (a) and poly (b) crystals produced from slow and rapid crystallization, respectively. Scale bar equals 50 μm. Time until homogeneous as a function of (c) dry crystal mass and (d) estimated surface area of a crystal. Each data point is an average of the corresponding data set, where each data set is made up of repeat measurements involving 8–15 droplets under identical environmental conditions. The uncertainties indicate the standard deviations in the average time, crystal mass (c) and surface area (d) of the population of individual particles within a given sample set.

The final crystal morphology depends on the crystallization RH and drying rate (Figure 3b,e), affecting properties such as the surface area, density, and shape of the dried particle. The relationship between the surface area or crystalline mass and the time for dissolution is explored in Figure 6. It may be expected that an increase in crystal mass or surface area increases the time taken for complete dissolution. Experiments were performed at an upper dissolution RH of 95% to ensure that the RH was not a limiting factor in the measured dissolution time of crystals of varying mass or surface area. To generate particles covering a range in dry mass, the starting concentration in the solution was varied (50–370 g/L) along with the starting radius of the droplets at generation. The pulse signal sent to the micro dispenser was varied to produce droplets in the radius range 20–32 μm. Overall, it was possible to achieve a range in estimated dry crystal mass of 2–28 ng.

In addition to varying the crystal mass, the crystals were formed at two different RHs, ∼2% and ∼40%, to control the morphology of the dried NaCl crystal and the surface area while keeping the dry mass constant. The surface area of particles formed under different drying conditions can be estimated from the number of crystals observed in the SEM images. Figure 6a,b confirms that formation of crystals is reproducible, and all crystals appear uniform in shape. When dried at lower RH, SEM images indicate that the polycrystalline particles have an average of 5 slabs per composite particle. The connection between each of the 5 crystals was on corners and edges only, and the total surface area of the polycrystal is taken as the sum of the estimated surface area of each inclusion crystal. From this information, it is possible to estimate the surface areas of mono and polycrystalline particles, μm^2^, with variation in initial starting solute concentration, droplet diameter, and RH. The estimated surface areas of the particles studied range from 500 to 4000 μm^2^. The relationships between particle surface area, mass, and dissolution time (the average time until a dissolving particle forms a homogeneous droplet at 95% RH) are reported in Figure 6b,c.

The data in Figure 6c support the hypothesis that there is a relationship between the crystal mass and total dissolution time (including the initial lag period). As the crystal mass increases the time taken to fully dissolve also increases. Crystal mass appears to be a good indicator of dissolution time with a clear correlation between the dissolution time and the crystal mass. Indeed, a similar correlation is observed between the dissolution time and surface area, Figure 6d. The surface area estimates are deduced from the SEM images in Figure 5. The data in Figure 6c are suggestive of a longer dissolution time for monocrystals than for polycrystals. However, the increase is marginal and within the uncertainty of the measurements.

Many APIs and excipients that are frequently used in the treatment of respiratory diseases deliquesce at an RH well above 90% (Table 1). In the case of lactose monohydrate, a commonly used drug carrying excipient, it has a DRH of 95%, only 4% lower than that of the lung. To put into perspective, Figure 5c indicates that an NaCl crystal will dissolve 2–3 times faster at >88% RH than 78% RH. Given that an average breath is ∼4 s, if the time and RH relationship were like that of NaCl, none of the excipients or APIs in Table 1 would dissolve prior to deposition. Partially or undissolved drug formulations have an impact on the subsequent pharmacokinetics and thus the efficiency of the drug delivery.^27^ The kinetic limitation induced by a difference between the water activity in the saturated solution and the RH of the gas phase should be taken into account when deposition fraction measurements of drug formulations. It should be noted that typically DPI starting formulations are produced using techniques such as spray drying. This results in the starting particles not being crystalline but rather being in an amorphous state. Meaning, the dissolution dynamics would be expected to be different for spray dried formulations compared to the crystalline structures explored here. Further study into the interplay between particle fabrication and subsequent dissolution dynamics is needed.

## Conclusions

Through controlling the drying kinetics (and subsequent morphology) of a simple crystalline NaCl particle, we demonstrate the ability to control the dissolution rate. We show that the rate of water evaporation during crystallization of NaCl affects the dried crystal morphology; rapid evaporation is more likely to lead to polycrystals and slow evaporation to monocrystals. Following this, we show that the difference in crystal morphology appears to have only a marginal impact on the dissolution time of crystalline aerosol particles, but a clear correlation between dissolution time and particle mass is observed. Additionally, the dissolution time is shown to depend on the RH of the gas phase. As the disparity between the RH and the DRH is reduced, i.e., as the RH becomes more comparable to the water activity in a saturated salt solution at an RH of 75%, the average time until dissolution increases. At an RH of 88% and above, the time until dissolution is not limited by the RH, and the dissolution and condensational growth are limited by gas diffusional transport. Finally, it is shown that there are additional limitations during dissolution compared with gas-phase diffusion limited condensation. A lag period of ∼5 s is observed by the dried crystal before any phase or size change is observed following introduction into a humid atmosphere.
